# Study of Thermal, Mechanical and Barrier Properties of Biodegradable PLA/PBAT Films with Highly Oriented MMT

**DOI:** 10.3390/ma14237189

**Published:** 2021-11-25

**Authors:** Joanna Ludwiczak, Stanisław Frąckowiak, Karol Leluk

**Affiliations:** Faculty of Environmental Engineering, Wrocław University of Science and Technology, 27 Wybrzeże Wyspiańskiego St., 50-370 Wrocław, Poland; stanislaw.frackowiak@pwr.edu.pl (S.F.); karol.leluk@pwr.edu.pl (K.L.)

**Keywords:** chain extender, films, montmorillonite, nanocomposites

## Abstract

In order to improve the properties of biodegradable polylactide (PLA), mixtures with polybutylene adipate-co-terephthalate (PBAT) were prepared. PLA is a bio-based and renewable biodegradable material, made from starch. PBAT is a biodegradable polyester for compostable film. In order to improve the composite properties, two types of additives were implemented via melt mixing, a chain extender (CE) and montmorillonite (MMT). CE was used as an interfacial modifier to enhance the adhesion between components. Montmorillonite is a widely studied clay added to polymer nanocomposites. Due to the lamellar structure, it improves the barrier properties of materials. PLA/PBAT films were oriented in the extrusion process and the amounts of filler introduced into the PLA/PBAT nanocomposites were 1.0, 3.0, and 5.0%. The improvement in the PLA barrier properties by the addition of PBAT and 5% of MMT was confirmed as the oxygen permeability decreased almost by a factor of 3. The addition of the biodegradable polymer, chain extender, montmorillonite, and the implemented orientation process resulted in a decrease in composite viscosity and an increase in the PLA crystallinity percentage (up to 25%), and the wettability tests confirmed the synergic behavior of the selected polymer blend.

## 1. Introduction

In recent years, more and more attention has been paid to environmental protection. Biodegradable polymers such as polylactide (PLA), poly(butylene succinate) (PBS), poly(butylene adipate-co-terephthalate) (PBAT), polycaprolactone (PCL), and thermoplastic starch have gained great popularity. PLA, representing a bio-based polyester, is produced from starch. It is a particularly important polymer; apart from its biodegradation properties, it comes from renewable sources and has a lower price compared to other biomaterials. Despite the advantages of PLA, its limitations in many applications are brittleness and low tensile strength, and in order to reduce these effects, blends with various biodegradable materials have been investigated. Therefore, in order to improve the properties of PLA without losing the biodegradability of the final product, many researchers have adopted PBS, PCL, PBAT, and thermoplastic starch as plasticizers. PBAT is a biodegradable polyester intended for the production of films.

Due to the immiscibility of PLA and PBAT, the challenge is to increase their compatibility with each other. Al-Itry et al. [1] prepared a thermally stable blend of PLA and PBAT with the addition of a chain extender named Joncryl ADR-4368 from BASF, which increased the viscosity and molecular weight of the PLA, improving the thermal stability and elasticity during melt processing due to the formation of branching chains, which is beneficial for films’ preparation. Other authors investigating a similar blend [2,3,4] confirmed that the change in morphology may have been due to a decrease in interfacial tension, possibly due to the formation of PLA/PBAT copolymers at the interface, and a change in the viscosity ratio favoring the presence of a chain extender. Other methods used include transesterification reactions [5,6], the use of peroxides that act as free radical initiators for in situ compatibility [7,8,9,10,11], and the addition of a triblock PLA/PBAT/PLA copolymer [12,13], acetyltributyl citrate [14,15]. Ma et al. [10] prepared in situ compatible PLA/PBAT blends in the presence of dicumyl peroxide (DCP) as a free radical initiator, which resulted in the formation of a PLA-g-PBAT copolymer. Zhang et al. [16] used glycide methacrylate as a processing agent for a PLA/PBAT blend. The authors achieved improved strength and storage modulus and better phase morphology. Recent reports describe the use of environmentally friendly additives in PLA/PBAT materials. Carbonell-Verdu et al. [17] recently reported the use of vegetable oil derivatives, epoxidized (ECSO) and maleinized cottonseed oil (MCSO), as a compatibilizing agent for PLA/PBAT blends. Other authors [18] successfully used an epoxidized cardanol-based prepolymer (ECP) generated from cashew nut shell liquid (CNSL) as a bio-based compatibilizer for PLA/PBAT blends.

Apart from the above additives, many researchers [19,20,21,22] have stated that layered silicates can act as compatibilizers in mixtures, having a positive effect on the morphological properties. Nanoclays are among the most studied nanomaterials. Montmorillonite (MMT) clay, which consists of platelets with an inner octahedral sandwiched between two silicate tetrahedral layers, is frequently used as a filler to produce polymer nanocomposites. The authors of [22] analyzed the interfacial tensions in the PLA/PBAT/MMT system and confirmed that MMT is located at the interface between PLA and PBAT, which stabilizes the phase morphology. This helps to improve the compatibility between PLA and PBAT. Other authors [23] introduced modified clay into PLA/PBAT to improve the properties of the blends. Hydrogen bonding was suspected from interactions between hydroxyl groups from clay interlayer galleries and PLA/PBAT blends. Nanoclay, thanks to its lamellar structure, can also improve the strength properties and constitute a barrier to gas permeability in polymer nanocomposites [24]. These characteristics are the result of the molecular interactions among the biopolymer matrix and clay surface [25]. The improvement of the mechanical properties can occur through strong interactions between the polymer and MMT groups [25,26]. The authors of the study [27] used OMMT nanoplates and investigated the barrier properties of PLA. Ethylene glycol diglycidyl ether (EGDE) was used for OMMT interlayer spacing control. It was found that the increased crystallinity of PLA improved the efficiency of the gas barrier in PLA/OMMT foil.

This work focused on PLA with the addition of PBAT, modified with both a chain extender (CE) and montmorillonite (MMT). Oriented films were produced from the prepared nanocomposites, which can be successfully used as fully biodegradable packaging with increased barrier properties. The influence of additives and the orientation process on PLA was investigated in terms of mechanical, thermal, rheological, and barrier properties.

## 2. Materials and Methods

### 2.1. Materials

Polylactide (PLA) Ingeo 2003D supplied by Nature Works LCC (Minnetonka, MN, USA), polybutylene adipate-co-terephthalate (PBAT), and Ecoflex F Blend C1200 (BASF) were used. PLA and PBAT (80/20 ratio) were blended, and the addition of 1.0 wt.% of chain extender (CE) Joncryl 4368 (BASF) was used. Nanocomposites were prepared using the PLA/PBAT mixture with montmorillonite Nanomer I28E (Sigma-Aldrich, St. Louis, Missouri, MO, USA), which contained 25–30 wt.% trimethyl stearyl ammonium. The particle size of the MMT filler given by the manufacturer was ≤20 μm. The amounts of filler introduced into the PLA/PBAT nanocomposites were 1.0, 3.0, and 5.0%.

### 2.2. Composite Preparation

**Figure 1:**
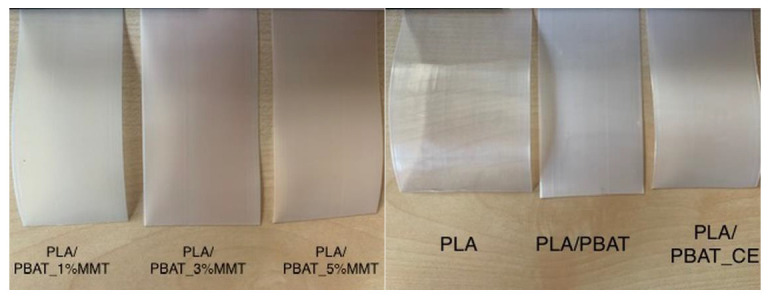
PLA, PLA blends, and PLA/PBAT with MMT films.

All materials were plasticized and blended using the Haake PolyDrive equipped with an internal mixer (from Thermo Fisher Scientific, Waltham, MA, USA), at a temperature of 180 °C and a rotor speed of 50 rpm. Mixing was concluded when the on-line measured torque reached equilibrium. The final materials were produced via extrusion molding (twin-screw extruder, CTW100 from Haake) with a sheet die (width of 150 mm) with a flexible die lip option that enabled the on-site adjustment of the sheet thickness as well as optimization of the diameter. The temperature profile of the extruder was 155–165–180–195 °C. Films were drawn and uniaxially oriented (Figure 1). A heated calender (40 °C) was used to form all the films with a draw ratio of approximately 3.

### 2.3. Mechanical Tests

The samples for mechanical tests were prepared using an injection molding machine (PROMA, Toruń, Poland) at a temperature of 190 °C. This method of sample preparation was selected instead of cutting directly from the obtained films in order to reduce the influence of low sample thickness, partial anisotropy, and similar phenomena that are related to the extrusion process.

Tensile properties (Young modulus, tensile strength, percentage strain at beak) were tested at a speed of 50 mm/min using a LLOYD LR10K machine (from Lloyd Instruments, Largo, FL, USA), following the ISO 527 standard. Samples of 4.0 mm by 2.0 mm were used for the study. Each measurement series consisted of 7 samples.

The measurement uncertainties were estimated differently for each of the tests. For the tensile test, 5–7 samples (dog-bone) were measured and the arithmetical mean was calculated. For each parameter (modulus, strength, strain), the mean value was calculated after the extraction of greatly differing experimental values. Error was calculated as standard deviation from the mean value.

### 2.4. Rheology Tests

Rheology tests at high shear rates (100–2300·s^−1^) at 190 °C were performed using the Rheo-tester 1000 capillary rheometer (from Goettfert, Buchen, Germany). The effect of adding mineral filler on the rheological properties of the PLA/PBAT blend was evaluated using a capillary die with a diameter of D = 0.5 mm and L/D = 40. No Bagley correction procedure was implemented due to the high L/D value. For the analyses, the results of apparent viscosity versus the shear rate were presented on double logarithmic scale. 

Melt rheology was performed twice for each material. As the output values were repetitive, it was assumed that the experimental error was no greater than 5%. For visual clarification of plots, corresponding error bars were not included on the plot.

### 2.5. DSC Measurements

Differential scanning calorimetry (DSC Q20, TA Instruments, Eden Prairie, MN, USA) was used to investigate the thermal properties of the materials. During the tests, characteristic phase transitions such as glass transition, melting, crystallization temperature, and degree of crystallization were determined. A thermal ramp was applied from −90 °C up to 200 °C at the rate of 10 °C min^−1^ under nitrogen flow. The cooling rate was set to −5 °C min^−1^. The percentage of crystallinity was calculated according to the following formula:

(1) $$\mathrm{Percentage}\;\mathrm{of}\;\mathrm{crystallinity}\;\left[\%\right]=\frac{\left[∆Hm\;–\;∆Hc\right]\;}{∆Hm{}^{\circ}\times 100\%}$$

where ∆*Hm* (J/g) is the melting enthalpy, ∆*Hc* (J/g) is the cold crystallization enthalpy, and ∆*Hm°* is the melting enthalpy of a 100% crystalline PLA reported to be equal to 93 J/g. DSC measurement was conducted once for each sample. Temperature peak position error was assumed to be 0.1 °C and enthalpy error was assumed to be 1 J/g (according to manufacturer’s note).

### 2.6. Surface Morphology and Oxygen Permeability

The morphology of all materials was characterized using scanning electron microscopy (VEGA3 LM, TESCAN, Warrendale, PA, USA). Samples were broken in liquid nitrogen and the fracture surface sputtered with gold prior to testing. During microscopic observations, a magnification of 2.5k× was used. 

Estimation of the oxygen permeation rate of thin films (ca. 100–200 µm thick) was performed using a MultiPerm analyzer (Extra Solution, Pieve Fosciana, Italy). The rectangular samples (10 cm × 10 cm) were placed in the measuring chamber and pressed by the apparatus fixture. A pure nitrogen and hydrogen (1%) mixture was used as a carrier gas, whereas technical-grade oxygen was the measuring one. OTR was measured in the following conditions: cell temperature 23 °C, humidity 50%RH. The international standard (ISO 15105-2:2003) was utilized to conduct the experiment with the highest possible accuracy.

The OTR experiment was repeated twice for each of the materials. Due to the repetitive behavior of the samples, it was not necessary to perform more experiments. OTR values presented on the plot were calculated as the arithmetical mean and corresponding standard deviation value for each mean.

Contact angle measurement was performed on pieces of foil strips, extracted by cutting from a large plate. Measurements were conducted using an SEE System goniometric apparatus. Three liquids (water, formamide, and didiodomethane) of analytical grade were utilized. The strip was large enough to place at least 5 (typically 7) drops of the measuring liquid. After capturing an image, the contact angle was estimated by a computer program and further calculations were applied. For each contact angle value, surface free energy (SFE) was calculated, based on which the arithmetical mean SFE was calculated together with the SFE standard deviation. This procedure was applied when calculating all contributions. For plot clarity purposes, error bars are not included, and numerical values are presented in the corresponding table.

## 3. Results

### 3.1. Mechanical Properties

The mechanical properties of the obtained materials are listed in Table 1. PLA is a rigid polymer with a Young’s modulus of approximately 2915 MPa and tensile modulus of 74.1 MPa, while PBAT is a significantly more flexible material. The Young’s modulus for PBAT is approximately 136 MPa and its elongation at break 508%, compared to 6.5% for PLA. The results obtained for the PLA/PBAT blend indicate that both polymers are incompatible. The addition of CE causes a decrease in the Young’s modulus and a slight increase in the percentage of elongation at break, which may indicate the improved miscibility of both polymers in the presence of a chain extender. The addition of the clay improves the miscibility of both components, as described by He et al. [22], especially with the addition of 5% MMT. The elongation at break of the PLA/PBAT/5%MMT composite increases to a value of approximately 43%. The value of Young’s modulus is reduced (1862 MPa) and the tensile strength increases to a value of 50.5 MPa for nanocomposites with the addition of 5% MMT. As stated by the authors, the FTIR spectrum shows that there exists a strong bond between MMT and single PLA or PBAT. Furthermore, MMT was found to be located in the interfacial region of the PLA/PBAT blend. Although keeping in mind the incompatible nature of both polymers, they have proposed an explanation for the increased elongation at break, stating that when the polymer is under forced deformation, holes develop in the interface of MMT and PLA and blunt the formulated cracks, which prevents the further expansion of the cracks. We believe that our findings can confirm the similar behavior of the investigated systems. However, the role of MMT as an active interfacial modifier in immiscible polymer blends is still a subject of research as it is not fully understood, particularly with regard to the observed toughening mechanism accompanying the material’s deformation.

### 3.2. Melt Rheology

**Figure 2:**
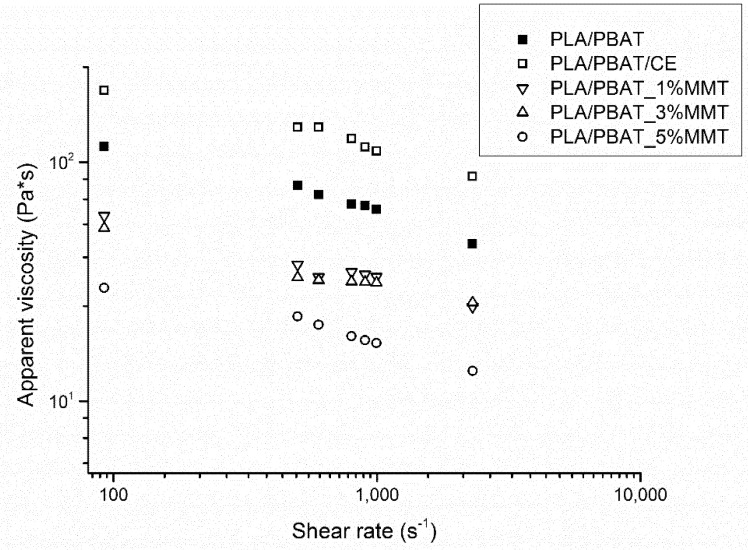
Apparent viscosity versus shear rate for PLA/PBAT blend and MMT-filled composites.

Apparent viscosity versus shear rate results for the obtained blend and composites with different filler content are presented in Figure 2.

One could expect an increase in viscosity with the addition of mineral filler; however, as can be observed in Figure 2, the viscosity tends to decrease with increasing filler content. Such behavior can be explained by anisotropic particles that induce their preferential orientation under high shear rates, as described by Gu et al. [28]. Due to shear thinning at higher filler loadings, the proposed orientation mechanism can describe the observed phenomena.

### 3.3. Thermal Properties 

The thermal results obtained for the PLA, PBAT, PLA/PBAT blends, and nanocomposites are presented in Table 2. Pure PLA is characterized by a glass transition temperature (Tg) of approximately 61.7 °C and a melting temperature (Tm) of 152.6 °C. The process of film orientation resulted in an increase in the degree of crystallinity to the value of 16%, and the occurrence of the cold crystallization temperature (Tcc = 127.5 °C). PBAT has a Tg of approximately −28 °C and Tm = 124.2 °C. The PLA/PBAT blend shows two signals assigned to glass transition (−30.3; 61.3 °C) and melting (110.5; 150.0 °C), which indicates the incompatibility of its components. Similar phenomena were observed in other studies related to PLA/PBAT blends [2,29,30]. There was no significant effect of CE on the thermal characteristics of the material. As the MMT content increases, the melting point shifts to a lower temperature, and this effect is more pronounced for the cold crystallization temperature. The orientation process as well as the addition of PBAT and MMT increase the percentage of PLA crystallinity. The addition of MMT results in a heterogeneous nucleation effect. The highest value (reaching up to 24–25%) was recorded for MMT addition (3; 5%) in the PLA matrix. The influence of MMT on increasing the degree of crystallinity has also been described in the literature [22,27].

### 3.4. Morphology

**Figure 3:**
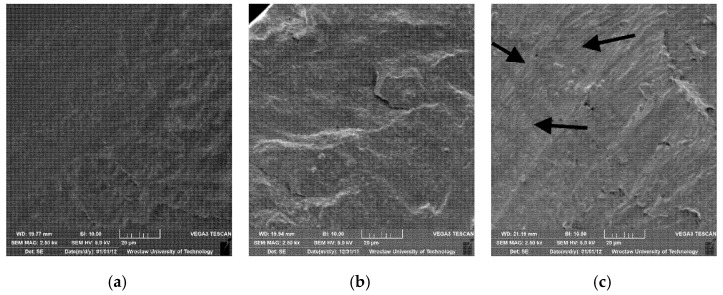
Microscopic images of selected materials: (**a**) PLA/PBAT blend; (**b**) PLA/PBAT_5%MMT blend parallel to the direction of extrusion; (**c**) PLA/PBAT_5%MMT blend perpendicular to the direction of extrusion (arrows mark the visible MMT structures).

The microscopic image of the PLA/PBAT blend showed no separate interphases of the two matrices, which indicates a partially miscible system (Figure 3a). 

The PLA/PBAT nanocomposites with the highest content of nanofillers (5%) broken parallel and perpendicular to the direction of extrusion are presented in Figure 3b,c. Observations of the fracture surfaces show good filler dispersion in the composites. The mechanical studies and obtained results also indicate that MMT was well-dispersed in the polymer matrix. Extended and oriented galleries of MMT in the polymer blend were obtained.

### 3.5. Oxygen Permeability

**Figure 4:**
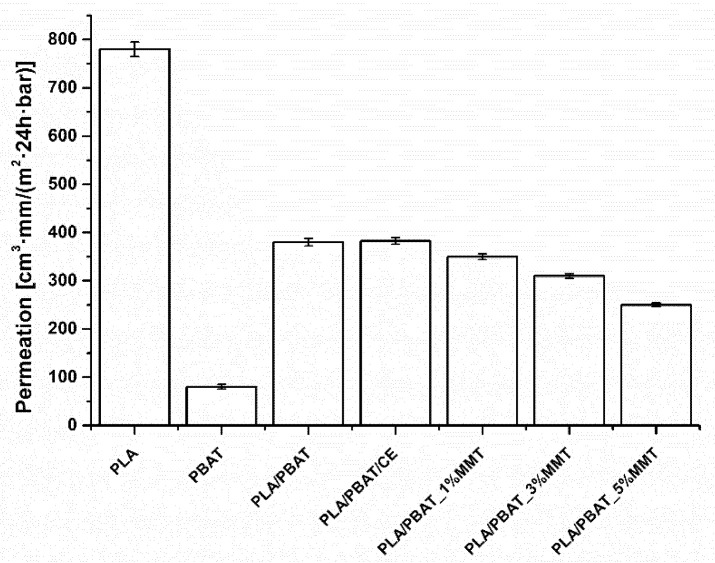
Oxygen permeability of PLA, PLA/PBAT blend, and PLA/PBAT/MMT nanocomposites.

PLA is one of the polymer plastics revealing a substantial level of oxygen and water vapor permeability. In terms of packaging, high gas permeation through the polymer membrane becomes an issue and has to be improved. The incorporation of fillers is the most commonly utilized practice but it is worth noting that the addition of a high-barrier polymer material periodically leads to a polymer blend with low gas permeation. In line with this concept, PBAT addition to PLA resulted in a reduced OTR by more than 50% (Figure 4). Neat PLA revealed an OTR level of 780 cc mm/m^2^ 24 h bar, whereas the PLA/PBAT blend yielded a value of around 350 cc mm/m^2^ 24 h bar. Substantial addition of montmorillonite resulted in a corresponding reduction in OTR, but the initial drop was not observed further.

An interesting fact is that the addition of a small (20%) portion of PBAT to the PLA matrix produces a blend with a non-proportional gain in oxygen barrier properties. Since gas permeation through polymer membranes is of a diffusive nature, this synergistic effect may also be treated as a result (and indicator) of a homogenous polymer system. Having a high distribution and dispersion rate of a low-permeation phase (PBAT), it is not necessary to load an additional portion of (more expensive) barrier material.

The addition of a chain extender to the blend did not significantly change the OTR, so the effect of CE can be neglected, contrary to MMT, which led to a further (slight) decrease in a measured parameter. The orientation of lamellar MMT reduced the OTR to 230 cc mm/m^2^ 24 h bar, which is four-times lower compared to unmodified PLA.

### 3.6. Wettability

The contact angle is an analytical measure of physical and chemical interactions between a measuring liquid and the characterized surface. As physical interactions are mainly restricted to the material’s surface area, the chemical part is related to much more complicated phenomena. The presence of polar groups or nucleophile or electrophile species in the main polymer chain to a high extent defines its hydrophilic or hydrophobic character. The situation becomes complicated when another polymer is introduced and/or a highly polar filler is added. Furthermore, physical treatment of the surface induces chemical changes in the surface area. Abrasive tools utilized during processing (or post-processing) may lead to sharp oxidation of the material. 

In this particular examination, all three samples were prepared in the same manner. Foil strips were prepared using a press molding process under the same conditions (temperature, time, and pressure), so it can be assumed that differences in surface area among the processed materials can be neglected. Observed differences in the measured contact angle values and thus surface free energy are then of a chemical nature. 

**Figure 5:**
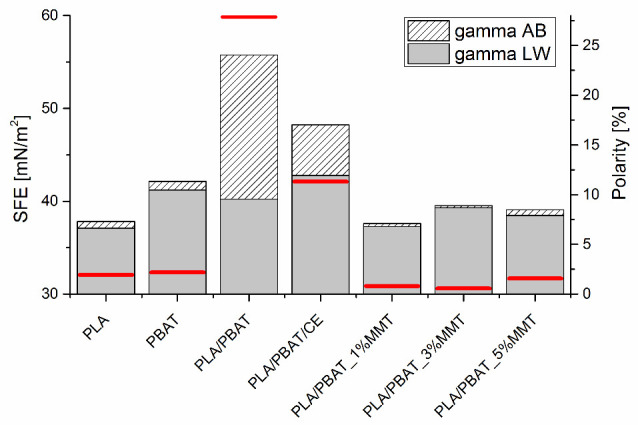
Surface free energy: Lifshitz–van der Waals (grey bars) and acid–base contribution (bars with diagonal lines). Red lines represent the sample’s polarity characteristic.

Figure 5 depicts the calculated surface free energy based on the van Oss–Good–Chaudhury model assumptions as well sample polarity (vertical lines). According to the model, total surface free energy consists of Lifshitz–van der Waals (gamma LW) and acid–base components (gamma AB), whereas polarity was calculated as the percentage contribution of the acid–base component to the total surface free energy.

Table 3 contains the calculated error values (standard deviation). As it can be clearly seen, the Lifshitz–van der Waals contribution slightly changed in all samples, generating the highest value for mixtures containing PBAT plastic. Similarly, the acid–base component remains almost unchanged in PLA and the three composites filled with different ratios of MMT. The PBAT sample demonstrates a higher Lifshitz–van der Waals component, compared to pure PLA, whereas a balance of both components remains at an unchanged level (polarity is comparable to that recorded in PLA sample). 

An interesting observation was made when introducing PBAT into the PLA matrix. This process resulted in a vast increase in the acid–base component and thus overall surface free energy value. The increase was almost 50% (when compared to pristine PLA) and two-times lower for the sample with the chain extender (PLA/PBAT/CE). Obviously, this situation is reflected in the polarity factor dependency, which indicated that the most polar samples were those containing PBAT, while MMT-filled composites behaved as non-polar systems.

The explanation for this lies in the chemical nature (polymer chain structure and composition) of all utilized polymer systems and the filling material.

**Figure 6:**
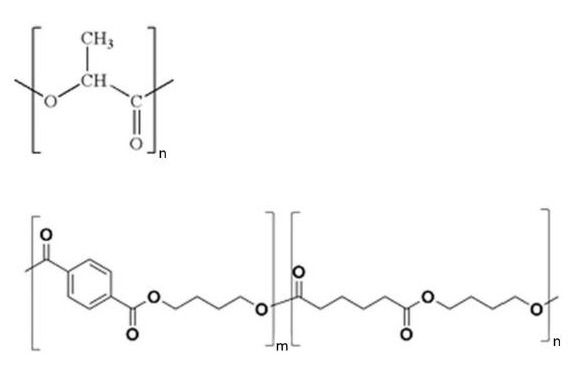
PLA (**top**) and PBAT (**bottom**) structural representation.

Referring to Figure 6, both PLA and PBAT are polyesters, consisting of carbon, oxygen, and hydrogen atoms in their chain. Table 4 contains their mass (mC, mH, mO) and molar ratios (nC, nH, nO). The calculation was performed under the assumption that in PBAT monomer m = n, and the molar masses are as follows: M_H_ = 1.00794 g/mol, M_O_ = 15.99936 g/mol, M_C_ = 12.066 g/mol.

In PBAT, when compared to PLA, the percentage contribution of carbon atoms to the mass and molar ratio is slightly higher. On the other hand, oxygen’s contribution (mass and molar) is pronounced in PLA. Oxygen atoms are highly electronegative (due to the presence of two free valence electron pairs), whereas hydrogen and carbon are not (most of the carbon atoms in both PLA and PBAT chains are four-bonded, sp^3^ hybridized ones). On this basis, it could be assumed that the higher oxygen atom ratio in PLA is related to the more polar nature of the polymer. This also explains why PBAT (having a markedly higher carbon to oxygen atom ratio) reveals a higher Lifshitz–van der Waals contribution of the total surface free energy, which is connected to non-polar interactions.

The addition of MMT particles led to a subsequent reduction in the acid–base component with increasing MMT ratio. This fact was also observed by other researchers [31] and was explained by the chemical nature of the filler. Organoclay, due to its mineral content, reveals moderate polar behavior [32].

The evident synergic behavior observed in both PLA/PBAT mixtures (with and without CE) resulting in an increasing acid–base component may be explained by the additional transesterification reactions acting in the system without a chain extender. This phenomenon was described in detail by Racha Al-Itry et al. [1], who examined similar systems. The addition of CE to the polymer mixture intensifies the chemical reactions among all constituents, making the system stiffer, with longer polymeric chains. This, in turn, diffuses the electron density, causing uneven distribution within the chain and making it less polar, which affects the acid–base component in the PLA/PBAT/CE sample. Similarly, the rising acid–base component with a constant Lifshitz–van der Waals contribution was also observed by Anita Ptiček Siročić et al. [33].

## 4. Conclusions

The influence of PBAT and various additives (MMT, CE) on the properties of PLA was presented. The preparation of blends may improve the polymer’s strength, while the addition of flexible matrices such as PBAT reduces the brittleness of PLA. It is also possible to significantly increase PLA’s viscosity by adding CE. Every additive investigated, as well as the film orientation process, had a positive effect, increasing the percentage of PLA crystallinity (up to 25%). The microscopic image showed that PLA/PBAT was partially miscible in the appropriate ratio. The melt blending and orientation process prompted the creation of a filler gallery in the PLA/PBAT films, consistent with SEM. The addition of PBAT to PLA improved the polymer’s barrier properties, while the highest observed effect in reducing gas permeability was obtained for the oriented PLA/PBAT film with the addition of 5% MMT.

All these modifications led to a subsequent improvement in the investigated materials’ properties, thus making this method feasible for a wide range of applications (i.e., packaging, low-temperature food packaging, agricultural mulch films).

## Figures and Tables

**Table 1:** Mechanical properties of selected materials.

Sample	Young’s Modulus (MPa)	Tensile Strength (MPa)	Percentage Strain at Break (%)
PLA	2915 ± 10.2	74.1 ± 1.4	6.5 ± 0.5
PBAT	136 ± 1.2	15.3 ± 1.1	508 ± 5.6
PLA/PBAT	2220 ± 4.5	56.2 ± 4.9	3.1 ± 0.3
PLA/PBAT_CE	1986 ± 4.2	51.3 ± 2.5	5.3 ± 0.2
PLA/PBAT_1%MMT	2396 ± 7.5	37.5 ± 2.1	7.8 ± 0.3
PLA/PBAT_3%MMT	2224 ± 5.4	43.1 ± 1.9	15.5± 1.1
PLA/PBAT_5%MMT	1862 ± 3.4	50.5 ± 3.2	42.9 ± 3.0

**Table 2:** DSC results.

Sample	T_g1_ (°C)	T_g2_ (°C)	T_m1_ (°C)	T_m2_ (°C)	T_cc_ (°C)	X_c_ (%)
PLA pure	-	61.7	-	152.6	-	3
PLA oriented	-	61.2	-	148.3	127.5	16
PBAT	−28.1	-	124.2	-	-	-
PLA/PBAT	−30.3	61.3	110.5	150.0	125.9	17
PLA/PBAT_CE	−31.2	61.8	108.9	151.4	127.7	15
PLA/PBAT_1%MMT	−30.3	60.8	106.6	150.9	122.6	23
PLA/PBAT_3%MMT	−31.2	60.0	106.5	149.7	117.6	25
PLA/PBAT_5%MMT	−31.7	60.1	106.6	148.7	114.1	24

**Table 3:** Calculation of error bars for SFE calculated values.

Sample	Gamma AB	Gamma LW	P
PLA	1.2	0.2	0.1
PBAT	1.8	0.3	0.2
PLA/PBAT	2.1	1.1	1.3
PLA/PBAT/CE	1.6	0.7	0.9
PLA/PBAT_1%MMT	1.5	0.5	0.8
PLA/PBAT_3%MMT	1.8	0.4	0.6
PLA/PBAT_5%MMT	1.7	0.3	0.5

**Table 4:** Calculation of mass and molar ratio in PLA and PBAT monomer.

% of Element	PLA	PBAT
mC	50.1	63.0
mH	5.6	6.7
mO	44.3	30.4
	100.0	100.0
nC	33.3	37.9
nH	44.4	48.3
nO	22.2	13.8
	100.0	100.0

## Data Availability

Not applicable.
